# Progress in Medicine

**Published:** 1899-10-14

**Authors:** 


### Progress in Medicine.

### DISEASES OF THE KIDNEY.

Waldeyer[1] admits the possibility of liquid being forced from the bladder into the ureters, and thence into the pelvis of the kidney, in spite of the valve at the mouth of the ureter. Even when the bladder is not over distended, if there is hypertrophy and an irritable condition, liquid may be thus forced backward. This has been often demonstrated in animals, and if the pressure exceeds that of the general circulation urine from the bladder may thus be driven through the kidney back into the renal vessels. This is, of course, of high importance with respect to uræmic conditions in surgical kidney. A possible instance of this in a case of gonorrhœa with pyelo-nephritis, where the gonococcus was found on a valve of the heart in pure culture, is recorded by H. Berg.[2] The patient suffered from primary gonorrhœa, followed by slight joint troubles and ulcerative pericarditis. In certain similar cases the joint troubles have been absent, but in some the gonococcus itself has been obtained from the joints. Councilman, Leyden, and others have found the gonococcus in the heart, as in this case. Berg believes that septic infection may be the result of the gonococcus alone.

Menzies[3] compares the results of Rose Bradford's and Tuffier's experimental excision of kidneys. Bradford found on excising less than two-thirds of the total kidney substance that the volume of the urine was permanently increased, while the urea was unaltered; and, if more than two-thirds were removed, both the volume and the urea were increased till life came to an end. Tuffier found no permanent alteration in urea or volume of urine after removal of successive portions, though these might in all be equal to the whole of the original kidneys through the hypertrophy which followed each operation. Another point in physiology may account for some of the œdema of Bright's disease. Reichel[4] remarks that normal transudation of tissue fluids depends on metabolism, and the activity of renal excretion, and he shows by experiment that this is upset in nephritis. Thus, he injected 50 cc. of saline fluid into various patients, and found that in renal cases a much longer time, even a week, would elapse before the swelling was absorbed.

**Cystic Kidney.**—T. Forbes[5] classifies cysts of the kidney as (*a*) cystic disease, congenital or acquired; (*b*) cysts in granular kidneys, either small multiple or large and single; (*c*) large serous cysts; (*d*) cystic growths. Similar groups occur in the liver. Cystic disease is the result of inflammation, and in the adult is produced by an over-growth of interstitial tissue compressing the tubules. It is really due to the same cause as granular kidney, where a few small retention cysts are present. In the liver the cysts are also due to inflammatory conditions akin to cirrhosis, the cysts arising from newly-formed bile ducts. Workman[6] records a case of adult cystic degeneration in a male aged 24. He had been ill eight months, but had no vomiting diarrhœa or headache. Hard masses were felt on either side of the abdomen. The urine was scanty, and contained blood and albumen but no casts. He died in a uræmic state. The kidneys weighed 67 and 65 ounces, and showed enormous numbers of cysts, but also active normal tissue and interstitial overgrowth, which, however, appeared to be secondary to the cyst formation. The liver was slightly enlarged, and was marked with numbers of small cysts. The persistence of normal kidney tissue shows how it is that these patients often live so long in fair health.

**Movable Kidney.**—German statistics show this occurs in about 1 in 250 persons. The *Medical Record*[7] recommends Noble's plan of examining patients in the upright position, unless a certain skill has been acquired in detecting loose kidneys. Watson Cheyne[8] records a case of movable third kidney, which existed on the right side of the spinal column just at the brim of the pelvis, having its own ureter and blood supply distinct from those of the other two normal organs. Edebohls[9] finds that in 80 or 90 per cent. of movable right kidneys which produce symptoms chronic appendicitis exists as a complication. Occasionally this develops into an acute attack.

He regards this condition as the most common form of appendicitis in women, and states that about one-half per cent. of all women have appendicitis without loose kidneys, while three and a-half per cent. have appendicitis with symptoms producing movable kidneys. He finds too that the associated pain is generally due to the appendicitis, and the latter trouble is a result of the movable kidney, not by direct pressure, but probably by interference with the blood supply through the superior mesenteric vessels. Nephropexy will thus cure the appendicitis if it is in the early catarrhal stage. Of this he has seen twelve instances, but if the disease has gone further both organs should be operated on at one and the same time.

A discussion of seventeen cases of movable kidney has appeared by A. P. Galloway,[0] who remarks that sometimes diagnosis is impossible without an exploratory incision. He lays stress on the dull aching pain increased by exertion, and the interference with the excretion of urine, as well as the presence of a movable tumour of special shape when this can be made out.

**The Causes of Nephritis.**—A. G. Nicholls[1] looks upon all forms of Bright's disease as various stages in the same process, which is generally due to infective agents. Chronic forms are most often the result of the action of the bacillus coli inducing interstitial inflammation and increase of the connective tissue. The point of invasion is through the gastro-intestinal tract, the liver and the mesenteric glands being a first line of defence. The malarial origin of certain cases is the subject of an analysis by Thayer[12] from Johns Hopkins Hospital. In 691 instances of malarial fever albumen was present in 46·4 per cent., and occurred most often in the æstivo-autumnal form. Acute nephritis is not an uncommon complication, the cases varying from one to three per cent., the greater proportion being in the irregular fevers. On the whole, malaria seems to originate a large amount of kidney disease, though the percentage is not so great as in some of the exanthemata.

The origin of granular kidney is carefully discussed by Samuel West[13] in the Lettsomian Lectures. He declares that he has never yet traced the kidney of acute nephritis into the small white kidney, that many sufferers from granular disease have never had acute nephritis, and others have had it only as an exacerbation during their chronic disease, and, finally, that cases of parenchymatous disease have been watched for many years without any indication of the supervention of granular kidney. On these and other grounds he considers that the latter has generally some other origin. Among symptoms he lays stress on thickened arteries in the early stages, and on albuminuria in the apparently healthy. "Physiological albuminuria," if more than a mere trace, is always pathological, and probably so when there is only a mere trace. However, other causes, such as heart or febrile diseases, must be searched for, and a distinction made between serum albumen and nucleo albumen. Retinitis, too, is an important symptom in later stages. Very rarely is it found in parenchymatous disease, and never elsewhere; but in granular kidney, if life lasts long enough, it is very frequent, and nearly always foretells an early and fatal termination. The white patches often radiating around the yellow spot are the most important part of the symptom, with which are conjoined hæmorrhages, exudations, and inflammatory charges.

Besides cardiac failure and hæmorrhages he directs attention to the frequency of aneurysms in the large vessels, a complication hitherto little suspected. Hæmaturia and epistaxis, as well as cerebral bleeding, are often met with. Chronic renal toxæmia, from perverted or increased metabolism, and acute uræmia from defective elimination, diarrhœa, renal asthma, headache, epileptiform convulsions apart from uræmia, and skin rashes are other signs which may direct our attention to this form of renal mischief. Besides rashes associated with œdema, there are others more or less spread over the whole body, such as pitysiasis rubra, and papular and erythematous forms. The presence of a universal eruption, together with albuminuria, is of most serious import. A paper on these eruptions in kidney disease[14] refers to instances of erythema leve, chronic pruritus, papular eczema, and urticaria. Lancaster's eight cases began with red maculæ, going on to papules, which spread from the extensor surfaces of the limbs over the whole body as a dermatitis exfoliativa. The writer records five instances of papular erythema followed by peeling, and sometimes accompanied by urticaria and petechiæ, as well as by uræmia and œdema. Simple uræmia is, he thinks, insufficient to account for these affections, and they may probably depend on the presence of some reducing substance, such as homogentesinic acid and the allies of alkapton, which has been observed in a similar instance. However, the true poisons in uræmia are unknown, whether they be products of perverted metabolism, toxins produced by microbial invasion, or the absence of internal secretions. Herringham, at the Pathological Society,[15] reported experiments showing that the toxicity of the urine is due to potash salts, and not to the auto-intoxicants put forward by Bouchard, but C. Turner remarked that in man the conditions are different, and the poisoning gradual; and Bradford pointed out that uræmia in disease differed widely from the form seen in excision of the kidneys. The latter[16] finds that the retention theory is quite inadequate to explain acute uræmia, which may occur when large quantities of urine and urea are being passed, while in obstructive or latent uræmia there is no coma, true convulsions, or dyspnœa. The methylene blue test has been put forward by Voisin as a measure of the toxicity of the urine. It will be remembered that Achard and Castaigne used it first as an indicator of renal permeability. Cabot,[17] like many others, has investigated it, but administers the blue usually by the mouth, and finds that excretion is delayed in granular kidney; while in other forms of nephritis it is as quick or quicker than in normal persons. The time of the first appearance of the dye is the most reliable result obtained. Other writers have noticed that no retardation takes place in albuminuria, due to congestion from cardiac disease. Cabot gives as little as three-quarters of a grain by the mouth, and finds the test a valuable means of diagnosis. Herter[18] thinks the results when given by the mouth less reliable, and holds that when the dye disappears from the urine in 36 hours we may probably say the kidneys are excreting from the blood the urinary constituents in a normal manner.

**Treatment.**—Bock[19] allows in arterio-sclerosis well-cooked meat and vegetable, except such as cause flatulence, dyspepsia, or ptomaines. Strong alcohol, coffee, or tea, fluids taken with meals, or exercise immediately after food are forbidden, as well as violent mental or physical exertion. S. West[20] also enjoins care in avoiding over-exertion and worry in granular kidney, and gives nitrate of pilocarpin by mouth two or three times a day. He finds that the usual sweating is rarely produced when this disease is present, yet the digestive and nervous symptoms are removed by the drug. Renal extract has not been much tried, but he finds good results from it; and it is curious to note that while taking it, pilocarpin brings about sweating as in normal patients. C. von Noorden[21] thinks there is no scientific ground for the distinction made between white and dark meats in the dietary of these patients. To restrict them to fowl, rabbit, and similar white meats only interferes with the appetite to little purpose. The highest percentage of nitrogenous extractives is actually found in white meat, as opposed to beef and other forms of red flesh. A more important point is the advantage of restricting the fluid taken, especially in advanced cases. He finds that where dilatation and cardiac asthma have begun a restriction of the fluid to at most 1¼ litres (44 ozs.) will often effect a surprising improvement. Even in earlier stages he is in favour of limiting the amount drunk. Moreover, from some long-continued experiments he finds that the elimination of the chief products of tissue-change is not decreased by this limitation, though the percentage of albumen in the diminished urine may mislead the observer. These results, so contrary to the received view of the value of flushing the tissues with water, will be more fully discussed in his promised paper. Another article by Barbier and Frankel[22] asserts that in healthy kidneys salicylate of sodium is a true diuretic, and when combined with caffeine it increases the flow to a high degree. On the other hand, Bouchard finds that diseased kidneys are much impeded by it.

Da Costa[23] praised lactate of strontium for acute nephritis in 15-grain doses every six hours. With this he employs pilocarpin, digitalis, and dry cupping. In uræmia Lemoine,[24] besides a milk diet and digitalis, prescribes pilocarpin by the mouth, and, when the respiration is difficult, large quantities of ether hypodermically and by the mouth. In comatose states he advocates bleeding, and the subsequent injection of one or two pints of saline solution.

A. B. Knowlton[25] also advises copious intravenous injections after bleeding. He has given as much as five pints at a time, and believes that even larger quantities are safe. He points out that the tension of the pulse is no guide to the amount desirable, for this is regulated after the first stimulation independently of the quantity. Yet the pulse and respiration will be quickened even apart from stimulation.

[1] Med. News, July 8. [2] Med. Rec., April 29. [3] Med. Chron., May. [4] Brit. Med. Jour., Mar. 3. [5] St. Bartholomew's Hosp. Rep., 1898, p. 181. [6] Glasgow Med. Jour., 1899, p. 379. [7] Med. Rec., Mar. 4. [8] Lancet, Jan. 28. [9] Postgraduate, Feb. [10] Glasgow Med. Jour., Jan. [11] Montreal Med. Jour., Mar. [12] Med. Chron., Mar. [13] Brit. Med. Jour., Feb. 11. [14] Brit. Jour. of Derm., July. [15] Brit. Med. Jour., Mar. 25. [16] Clin. Jour., Jan. 25. [17] St. Paul Med. Jour., Feb. [18] Practitioner, Mar. [19] Schmidt's Jahrbücher, Vol. 4, p. 30, 1899. [20] Brit. Med. Jour., Mar. 11. [21] Inter. Med. Mag., May. [22] Med. Bulletin, June. [23] New York Med. Jour., Jan. 28. [24] Treatment, Feb. 4. [25] Charlotte Med. Jour., Feb.

